# B-cell acute lymphoblastic leukemia and lymphoblastic lymphoma with p190 *BCR::ABL1* transcript: a case report

**DOI:** 10.25122/jml-2025-0020

**Published:** 2025-06

**Authors:** Budiono Raharjo, Catherine Keiko Gunawan, Stephani Linggawan, Siprianus Ugroseno Yudho Bintoro, Anton Sumarpo

**Affiliations:** 1 Faculty of Medicine, Universitas Wijaya Kusuma Surabaya, Surabaya, Indonesia; 2 Faculty of Medicine, Maranatha Christian University, Bandung, Indonesia; 3 Department of Internal Medicine, Faculty of Medicine, Universitas Airlangga -Dr. Soetomo General Hospital, Surabaya, Indonesia; 4 Department of Clinical Pathology, Faculty of Medicine, Maranatha Christian University, Bandung, Indonesia

**Keywords:** B-cell lymphoblastic lymphoma, acute lymphoblastic leukemia, *BCR::ABL1*

## Abstract

Lymphoblastic lymphoma (LBL) is a rare and aggressive lymphoblastic neoplasm, accounting for approximately 2% of non-Hodgkin lymphoma cases. Despite sharing clinical and morphological similarities with acute lymphoblastic leukemia (ALL), LBL is characterized by distinct genetic abnormalities. Due to the ambiguity surrounding treatments, the prognosis for LBL remains poor, with complete remission rates between 40-58% and 5-year disease-free survival rates between 36-70%. We present a case of a 42-year-old man diagnosed with B-acute lymphoblastic leukemia (B-ALL)/lymphoblastic lymphoma (LBL). The diagnosis was challenging due to the rarity of the condition and the overlapping features of LBL and ALL. This case report highlights the predominance of lymphoblasts and the presence of the p190 (e1a2) *BCR::ABL1* transcript, which is frequently associated with poor prognostic outcomes in lymphoblastic malignancies. The coexistence of both B-ALL and LBL underscores the necessity of a comprehensive understanding of the diagnostic approach, which is essential for optimizing treatment strategies and improving prognosis.

## INTRODUCTION

Lymphoblastic lymphoma (LBL) is a rare and aggressive form of B- or T-cell lymphoblastic neoplasm, constituting less than 10% of acute lymphoblastic leukemia and 2% of non-Hodgkin Lymphoma cases [1,2]. The World Health Organization (WHO) classifies LBL as closely related to ALL due to their overlapping characteristics [3,4]. While both conditions may exhibit distinct clinical features — mediastinal and/or soft tissue masses and extensive bone marrow infiltration in ALL—specific genetic abnormalities serve to differentiate the two disorders [4-6]. Notably, LBL frequently exhibits additional chromosomal abnormalities, such as trisomy or tetrasomy, which are less frequently observed in ALL. Moreover, cross-lineage rearrangements, particularly immature immunoglobulin heavy chain (IgH) rearrangements in T-LBL and TR gene rearrangements in B-LBL, have been observed [1]. To date, definite treatment for LBL remains elusive, resulting in an unsatisfactory outcome with complete remission rates between 40-58% and 5-year disease-free survival rates ranging from 36-70% [7-9]. Interestingly, T-LBL is more prevalent than B-LBL, with an incidence ratio of 9:1 [1]. In this article, we report an atypical B-LBL case in a 42-year-old man with p190 (e1a2) *BCR::ABL1* transcript, highlighting its unique characteristics and implications for diagnosis and treatment.

## CASE PRESENTATION

A 42-year-old male patient was admitted to the hospital with intermittent fever and shoulder pain, persisting for 4 days. He denied any history of cough, runny nose, diarrhea, hypertension, diabetes mellitus, and previous blood transfusion. Physical examination revealed a low-grade fever (38.0^º^C) and signs of anemia marked by anemic conjunctivae. Moreover, multiple well-defined, mobile masses in the abdomen, each measuring between 1 and 1.5 cm in diameter, were discovered.

The laboratory examination showed normochromic normocytic anemia with a hemoglobin of 9.2 g/dL (normal range: 13.0–18.0 g/dL), mean corpuscular volume of 87 fL (normal range: 80.0–100 fL), mean corpuscular hemoglobin of 30 pg (normal range: 27.0–32.0 pg), mean corpuscular hemoglobin concentration of 34% (normal range: 27.0–31.0 %). The erythrocyte count was markedly low at 3.12 million/µl (normal range: 4.7–6.1 million/µl), with a red cell distribution width (RDW) of 17.8%. Furthermore, the patient exhibited leukocytosis (19.5x10^3^/μL) and thrombocytopenia (36x10^3^/μL), along with a reticulocyte count of 0.6%, indicating ineffective erythropoiesis.

**Figure 1:**
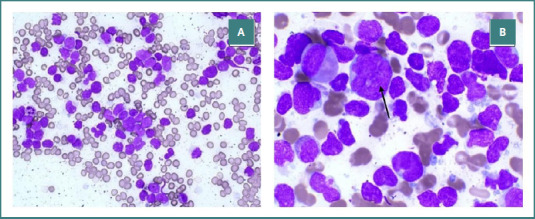
Pathological image of bone marrow aspiration. A, heterogeneous blast cells in 10x magnification. B, blast cell in 40x magnification, indicated by the black arrow.

The histological examination of the biopsied abdominal masses revealed a proliferative tumor characterized by round nuclei, coarse and indented chromatin, increased mitotic figures, and narrow cytoplasm arranged in uniform sheets with invasive stromal connective tissue and surrounding fat. The immunohistochemical studies showed positive expression of CD45, Ki-67, TdT, CD79a, and CD19, markers indicating the presence of B-cell lineage. However, it was negative for both CD3 and CD20, which are typically associated with T-cells. To further clarify the nature of the abdominal masses, a bone marrow aspirate was performed. The aspirate revealed hypercellularity, predominantly by lymphoblasts, which accounted for 94% of all cells examined (Figure 1AB).

Furthermore, immunophenotypic analysis confirmed the presence of CD34+, CD19+, CD79a+ and HLA-DR+, further substantiating B-cell origin, while showing negative CD3-, CD5-, CD20- and CD13- (Table 1 and Supplementary Figure).

Supplementary Figure

**Table 1:** Immunophenotyping and immunohistochemistry results confirm B-cell lineage

**Immunophenotyping**	CD34+, CD79a+, CD19+, HLA-DR+, CD19+, CD3-, CD5-, CD13-
**Immunohistochemistry**	CD45+, CD79a+, Ki67+, TdT+, CD3-

To fully understand the nature of the disease, a mutational profile was conducted, revealing the presence of the p190 (e1a2) *BCR::ABL1* transcript, resulting from a t(9;22) translocation, which is frequently associated with poor prognostic outcomes in lymphoblastic malignancies. Taken together, our findings confirmed the diagnosis of B-acute lymphoblastic leukemia/lymphoblastic lymphoma.

The chemotherapeutic treatment was initiated for B-acute lymphoblastic leukemia /lymphoblastic lymphoma with 10 cycles of imatinib-based chemotherapy. Following induction chemotherapy, a bone marrow aspiration demonstrated normal cellularity with decreased erythropoiesis and increased granulopoiesis without any identified lymphoblasts, indicating complete remission. However, a follow-up bone marrow aspiration revealed a relapse of B-ALL. Unfortunately, the patient failed to respond to further chemotherapy and succumbed to the disease 6 months later.

## DISCUSSION

B lymphoblastic lymphoma is a type of non-Hodgkin lymphoma that originates from lymphoblasts, accounting for approximately 10% of all lymphoblastic lymphoma cases. Unlike ALL, LBL typically has limited bone marrow involvement [1]. This report highlights a case of B-LBL in an adult associated with the uncommon p190 (e1a2) *BCR::ABL1* transcript, which is more commonly found in children [10]. This case involved a 42-year-old male patient who presented with multiple masses in the abdomen. This clinical manifestation aligns closely with B-LBL, a condition characterized by the proliferation of B-cell lymphoblasts, which manifest primarily in the lymph nodes and extranodal sites [11]. The resemblance in presentation underlined the findings reported by Kim *et al*., as both patients presented extranodal masses [12]. These traits were presumed to result from heightened angiogenesis, which plays a critical role in the initiation and progression of solid tumors as well as hematological malignancies [13].

Upon further examination of the laboratory findings, we noted significant anemia, leukocytosis, and thrombocytopenia, which reinforced our suspicion of acute lymphoblastic leukemia or lymphoblastic lymphoma [12]. While anemia and leukocytosis are common occurrences, thrombocytopenia observed in our case differs from previous reports; several studies suggest that platelet counts generally remain normal in ALL/LBL [12]. This discrepancy may stem from inflammatory response triggered by B-cells, with elevated pro-inflammatory cytokines such as interleukin-6 (IL-6), tumor necrosis factor-alpha (TNF-α), IL-1, and interferon-gamma (IFN-γ) [14]. These mediators are predicted to play a role in the suppression of erythropoietin, thus contributing to anemia [14]. Additionally, mutations in *ANKRD26, RUNX1*, and *ETV6* may predispose patients to thrombocytopenia by disrupting normal megakaryocyte function, significantly affecting platelet production [15].

The diagnostic investigation transitioned beyond blood work to the critical domain of immunohistochemistry. Analysis of the abdominal masses biopsy demonstrated positive staining for vital B-cell markers, including CD45, Ki67, TdT, CD79a, and CD19. These findings not only confirmed the presence of B-cell lineage but also aligned with a previous study [16]. Collectively, these results solidify our diagnosis, clearly indicating B-LBL.

Observation of the bone marrow further illuminated the case. Bone marrow aspiration revealed hypercellularity, with 94% of the examined cells comprising lymphoblasts. Our result contrasts with prior findings in lymphoblastic lymphoma, where bone marrow involvement is typically less than 25% [17]. The predominance of blast cells in this case supports the presence of acute lymphoblastic leukemia, thus affirming our conclusion that the patient had B-acute lymphoblastic leukemia/lymphoblastic lymphoma [1].

In most cases of lymphoblastic lymphoma, the diagnosis cannot be established solely based on clinical features. A definitive diagnosis requires comprehensive immunophenotypic analysis, which, in this case, revealed marked B-cell proliferation in the bone marrow and lymph node involvement [1]. The immunophenotypic profile showed strong expression of B-lineage markers, including CD34, CD79a, and CD19, consistent with previously reported cases [2,18]. While B-cell markers are typical findings in B-LBL, our case demonstrated aberrant expression of the T-lineage-associated marker HLA-DR.

The mutational profile of lymphoblastic lymphoma frequently overlaps with those found in chronic myeloid leukemia as well as B-cell acute lymphoblastic leukemia. In the present case, the genetic findings closely resembled those described by Li *et al*., with both cases exhibiting the *BCR::ABL1* fusion gene, specifically the p190 (e1a2) transcript arising from a t(9;22) translocation [19]. This cytogenetic abnormality produces a fusion protein that activates tyrosine kinase, leading to enhanced proliferation, differentiation, and survival of leukemic cells [20]. Moreover, patients expressing the p190 isoform typically exhibit a higher tyrosine kinase activity compared to those with the p210 isoform, resulting in a more aggressive disease progression [20,21].

The treatment of lymphoblastic lymphoma often follows regimens traditionally used for non-Hodgkin lymphoma. However, these approaches are associated with poor outcomes, with reported a complete remission rate of approximately 58% and a 5-year disease-free survival rate of 36%. In contrast, regimens designed for acute lymphoblastic lymphoma have demonstrated improved long-term outcomes, with a complete remission rate ranging from 55% to 100% and a 5-year disease-free survival rate ranging from 45% to 65%. Given the high risk of relapse, hematopoietic stem cell transplantation is commonly utilized as a consolidative strategy, either during the first remission or in cases of relapse. However, its precise role and prognostic indicators for patient selection remain unclear [22].

Our case had an aggressive and rare form of ALL/LBL, which typically requires intensive multi-drug leukemia chemotherapy protocols. Imatinib has proven to be an effective first-line treatment for adult Ph+ ALL when given concurrently with chemotherapy. Combining imatinib with intensive chemotherapy during remission induction and consolidation has shown high rates of complete remission, with morphologic remission achieved in 95-100% of cases and molecular remission in approximately 50% of cases. These improved outcomes have led to the combination of imatinib and chemotherapy becoming the standard of care for Ph+ ALL [23]. Unfortunately, due to the patient’s inability to receive chemotherapy following relapse, the patient succumbed to the disease 6 months after achieving complete remission.

## CONCLUSION

This case report delineates an uncommon characteristic associated with B-cell acute lymphoblastic lymphoma, particularly in the context of the p190 (e1a2) *BCR::ABL1* transcript. Future studies are necessary to deepen our understanding of the molecular pathways associated with different genetic profiles in B-LBL/ALL. Despite promising results for patients treated with a combination of both imatinib and chemotherapy, the search for improved treatment strategies continues to be imperative.

## Data Availability

Data supporting this research article are available from the corresponding author upon reasonable request.
