# Supplementary material for: B-cell acute lymphoblastic leukemia and lymphoblastic lymphoma with p190 BCR::ABL1 transcript: a case report
Source: J Med Life. 2025 Jun;18(6):600–3. doi: 10.25122/jml-2025-0020 (PMC12314840; doi:10.25122/jml-2025-0020)
Supplement: Supplementary file 1 [file JMedLife-18-600-s001.pdf]

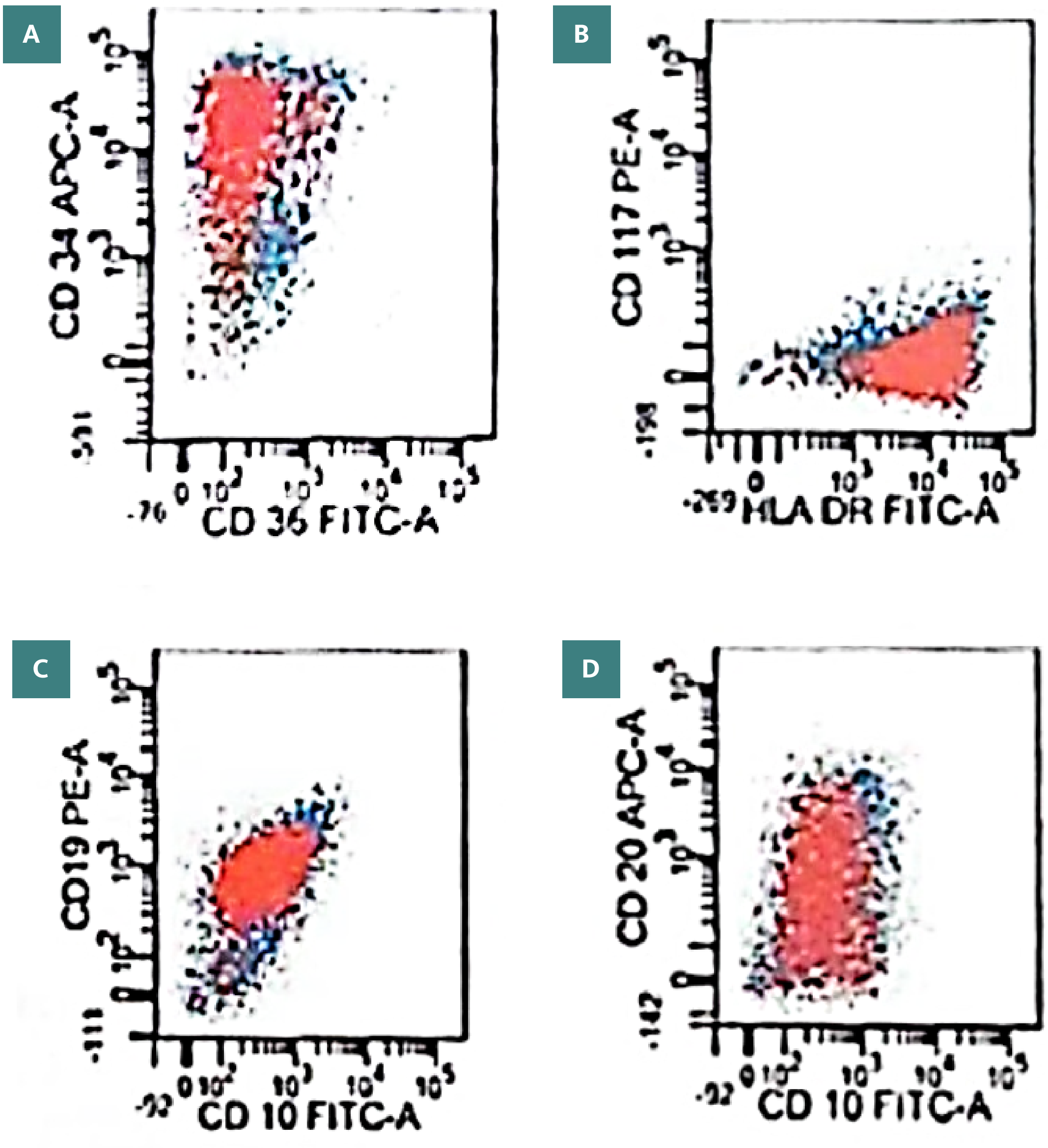

Immunophenotyping dot plots illustrating the B-cell lineage profile in lymphoblastic lymphoma. A, CD34/CD46 plot showing a cluster with strong CD34 and a low CD36 expression. **B,** CD117/HLA-DR plot showing a cluster with strong HLA-DR and a low CD117 expression. **C,** CD19/CD10 plot showing a cluster with strong CD19 and CD10 expression. **D,** CD20/CD10 plot showing a cluster with strong CD20 expression.

© 2025 by the authors. This article is an open access article distributed under the terms and conditions of the Creative Commons Attribution (CC BY 4.0) license.
